# Supplementary material for: A high-resolution mRNA expression time course of embryonic development in zebrafish
Source: eLife. 2017 Nov 16;6:e30860. doi: 10.7554/eLife.30860 (PMC5690287; doi:10.7554/eLife.30860)
Supplement: Supplementary file 6. [file elife-30860-supp6.zip › biolayout-clusters-files/Cluster137-genes.html]

Cluster137


# Cluster137: Genes

| Ensembl ID | Gene Name | Chr | Start | End | Biotype |
| --- | --- | --- | --- | --- | --- |
| ENSDARG00000043843 | akap7 | 20 | 2402637 | 2462287 | protein\_coding |
| ENSDARG00000027572 | as3mt | 1 | 30974100 | 30990307 | protein\_coding |
| ENSDARG00000005112 | cdh17 | 16 | 26900740 | 26929356 | protein\_coding |
| ENSDARG00000069752 | ckba | 13 | 15551083 | 15571680 | protein\_coding |
| ENSDARG00000089043 | ptpn6 | 16 | 31765421 | 31801373 | protein\_coding |
| ENSDARG00000055835 | slc26a6 | 8 | 26248961 | 26269367 | protein\_coding |
| ENSDARG00000042737 | slc52a3 | 8 | 28433888 | 28442758 | protein\_coding |
| ENSDARG00000039064 | st3gal7 | 13 | 40590429 | 40600975 | protein\_coding |
| ENSDARG00000041554 | unc93a | 20 | 43481951 | 43492397 | protein\_coding |
